# Chimeric Antigen Receptor T‐Cells in Myasthenia Gravis: Advances, Safety Challenges, and Future Directions

**DOI:** 10.1002/mus.70222

**Published:** 2026-04-10

**Authors:** Tobias Hegelmaier, Alexander Duscha, Christiane Desel, Sarah Stassen, Tim Krzemien, Ann‐Katrin Hennemann, Vaia Pappa, Aiden Haghikia

**Affiliations:** ^1^ Department of Neurology and Clinical Neurophysiology Hannover Medical School Hannover Germany

**Keywords:** autoimmune neuromuscular disorders, CAR T‐cell therapy, CD19/BCMA/CAAR T‐cells and B cell depletion, myasthenia gravis

## Abstract

This review examines the emerging application of chimeric antigen receptor (CAR) T‐cell therapy in myasthenia gravis (MG), with emphasis on safety, efficacy signals, and future therapeutic potential in treatment‐refractory disease. A comprehensive literature search was conducted across PubMed, medRxiv, bioRxiv, and Google Scholar for studies published between January 1, 2010 and July 1, 2025, using the terms “CAR T‐cell,” “chimeric antigen receptor,” and “myasthenia gravis.” Eligible reports included clinical trials, case reports, case series, and mechanistic studies describing CAR T‐cell therapy outcomes in MG. Across five independent case series, seven patients receiving CAR T‐cells targeting CD19, BCMA, or bispecific antigens showed consistent clinical improvement. Peak expansion occurred 8–22 days postinfusion, with persistence of 28 to ~180 days. Despite small cohorts, safety in CAR T‐cell therapy was favorable versus oncology, with mainly Grade 1–2 cytokine release syndrome, rare immune effector cell‐associated neurotoxicity, and no severe or life‐threatening events. Seven registered trials (> 260 patients) are expected to conclude between 2026 and 2029. Overall, CAR T‐cell therapy shows substantial promise for treatment‐refractory MG, potentially offering greater durability than existing therapies with an acceptable safety profile. Nevertheless, current evidence is restricted to small, uncontrolled studies, and long‐term efficacy, durability, and optimal patient selection require validation in larger controlled trials.

**Abbreviations**
AChR: acetylcholine receptor
ACPA: anti‐citrullinated protein antibody
B cells: B lymphocytes
BCMA: B‐cell maturation antigen
CAAR T: chimeric autoantibody receptor T‐cell
CAR T: chimeric antigen receptor T‐cell
CD19: cluster of differentiation 19
CD20: cluster of differentiation 20
CMV: cytomegalovirus
CRISPR: clustered regularly interspaced short palindromic repeats
CRS: cytokine release syndrome
CTLA‐4: cytotoxic T‐lymphocyte‐associated protein 4
DLBCL: diffuse large B‐cell lymphoma
EBMT: European Society for Blood and Marrow Transplantation
EULAR: European Alliance of Associations for Rheumatology
FcRn: neonatal Fc receptor
GM‐CSF: granulocyte‐macrophage colony‐stimulating factor
HB: hemoglobin
ICANS: immune effector cell‐associated neurotoxicity syndrome
IL‐6: interleukin 6
ITAM: immunoreceptor tyrosine‐based activation motif
IVIG: intravenous immunoglobulin
LAG‐3: lymphocyte‐activation gene 3
LEMS: Lambert–Eaton myasthenic syndrome
LRP‐4: lipoprotein receptor‐related protein 4
MG: myasthenia gravis
MG‐ADL: myasthenia gravis activities of daily living
MGC score: myasthenia gravis composite score
MGFA: Myasthenia Gravis Foundation of America
mTOR: mechanistic (mammalian) target of rapamycin
MuSK: muscle‐specific kinase
PD‐1: programmed cell death protein 1
QMG: quantitative myasthenia gravis score
TALEN: transcription activator‐like effector nucleases
T‐cells: T lymphocytes
TIGIT: T‐cell immunoreceptor with Ig and ITIM domains
TIM‐3: T‐cell immunoglobulin and mucin‐domain containing‐3
TNF‐α: tumor necrosis factor alpha
TRAC: T‐cell receptor alpha‐chain

## 1 Introduction

Myasthenia gravis (MG) is mediated by autoantibodies targeting components of the neuromuscular junction, including acetylcholine receptors (AChR), lipoprotein receptor‐related protein 4 (LRP‐4), agrin, and muscle‐specific kinase (MuSK). These autoantibodies are produced by autoreactive B cells or plasma cells, which play a pivotal role in MG pathogenesis by perpetuating the autoimmune response and driving disease progression [1, 2, 3]. The central role of B cells in MG has made them a primary therapeutic target. Despite major advances in immune therapies, approximately 10%–20% of patients remain refractory to standard treatments, highlighting an unmet clinical need for novel interventions [1, 4, 5].

B cell‐depleting monoclonal antibodies have significantly advanced the treatment of MG. Rituximab, an anti‐CD20 antibody, has demonstrated efficacy in reducing disease severity by depleting mature B cells and attenuating autoantibody production. This effect is particularly pronounced in MuSK‐positive MG patients, where rituximab has shown sustained clinical benefits [2, 3]. More recently, inebilizumab, an anti‐CD19 monoclonal antibody, has emerged as a promising therapy by targeting a broader spectrum of B cell populations, including plasmablasts and short‐lived plasma cells [6, 7, 8]. These advances underscore the potential of B cell‐directed therapies in modulating autoimmune processes underlying MG while also revealing limitations such as incomplete depletion of pathogenic subsets, insufficient tissue penetration, and adverse effects [4, 9]. Additionally, complement inhibitors (e.g., eculizumab, ravulizumab) and neonatal Fc receptor inhibitors (e.g., efgartigimod, rozanolixizumab) expand therapeutic options by reducing complement‐mediated damage or accelerating IgG clearance [10, 11].

Beyond CD20/CD19 depletion, direct targeting of antibody‐secreting cells has emerged as a mechanistically plausible escalation strategy in severe, therapy‐refractory MG. Proteasome inhibition with bortezomib can deplete plasma cells and plasmablasts and has been reported to induce rapid clinical improvement with concomitant reduction in MuSK antibody titers in a severe refractory MuSK‐positive MG case [1, 7]. Similarly, daratumumab (anti‐CD38) depletes CD38‐expressing long‐lived plasma cells and has been used as rescue therapy in a small neurology case series that included a patient with highly refractory (seronegative) MG, showing clinically meaningful improvement (QMG reduction) alongside broad immunoglobulin and autoantibody decreases, albeit with relevant infectious and hematologic risks in heavily pretreated patients [12].

Bispecific CD19 × CD3 T‐cell engagers provide an off‐the‐shelf strategy to redirect endogenous T cells toward B‐lineage cells. Off‐label use of blinatumomab in two patients with severe refractory generalized MG resuted in rapid and sustained clinical improvement with only low‐grade cytokine release syndrome reported [13]. The long‐term efficacy and optimal treatment schedules of T‐cell engagers in MG remain undefined.

In parallel, autologous hematopoietic stem cell transplantation (HSCT) has been pursued as a “high‐intensity” immune reset strategy in selected, otherwise treatment‐refractory autoimmune diseases and is referenced in contemporary expert recommendations for innovative cellular therapies. However, its role in MG remains exceptional, given the nontrivial treatment‐related morbidity and mortality as well as the limited MG‐specific evidence, since HSCT entails broad myelo‐ and immunoablation with prolonged immunosuppression. Accordingly, HSCT is best regarded as a rarely used salvage option rather than a scalable or competing alternative to CAR T‐cell therapy in MG [8].

Chimeric antigen receptor (CAR) T‐cell therapy has emerged as a potentially transformative approach in immunotherapy. Originally developed for hematological malignancies, CAR T‐cells are engineered to target specific antigens on diseased cells [14]. In autoimmune diseases, CAR T‐cells targeting CD19 and B‐cell maturation antigen (BCMA) have shown promise in preclinical clinical studies in MG [3, 15, 16]. Anti‐CD19 CAR T‐cells effectively eliminate a broad spectrum of B cells, while anti‐BCMA CAR T‐cells specifically target plasma cells resistant to conventional treatment [3, 17, 18]. These advancements suggest that CAR T‐cell therapy could provide durable immunomodulation by eradicating autoreactive B cell populations and lead to a so called *immune reset* [19].

Recent reports have demonstrated that CAR T‐cell therapy can achieve significant reductions in autoantibody levels and clinical improvement in systemic lupus erythematosus (SLE), and more recently in MG [3, 15, 19, 20, 21]. However, the findings in MG remain preliminary, with limited data on long‐term efficacy and safety profiles [3, 22]. The potential for severe adverse events, such as cytokine release syndrome (CRS), immune effector cell‐associated neurotoxicity syndrome (ICANS) or prolonged cytopenia, necessitates cautious evaluation before broader clinical implementation [23, 24]. The largest clinical experience with CAR‐T cell therapy in MG to date is provided by the prospective MG‐001 trial evaluating the RNA‐engineered anti‐BCMA CAR‐T product Descartes‐08 in 14 patients with generalized MG [16]. This multicenter phase 1b/2a study represents a substantive advance over previously reported case series by employing standardized manufacturing, uniform dosing, and systematic outcome assessment without lymphodepleting chemotherapy. Clinically meaningful improvements were observed across multiple validated endpoints (Myasthenia Gravis Activities of Daily Living [MG‐ADL], Quantitative Myasthenia Gravis Score [QMG], Myasthenia Gravis Composite [MGC], and Myasthenia Gravis Quality of Life 15‐item Revised [MG‐QoL15r]). Importantly, no cytokine release syndrome, immune effector cell‐associated neurotoxicity syndrome, prolonged cytopenias, or serious infections were observed, indicating a favorable acute safety profile compared with DNA‐integrating CAR‐T approaches [16].

Extended follow‐up of the MG‐001 cohort provides the most informative long‐term data currently available for CAR‐T therapy in MG. Sustained clinical benefit was observed for up to 9–12 months in the majority of patients receiving six weekly infusions, with most maintaining clinically relevant improvement at 1 year. Importantly, waning responses were not associated with cumulative toxicity and could be rapidly recaptured upon retreatment, suggesting reversible pharmacodynamic effects rather than permanent immune ablation [25]. Preserved immunoglobulin levels, maintained vaccine titers, and the absence of delayed infectious complications further distinguish this approach from long‐lasting B‐cell‐depleting strategies [25, 26, 27].

This review aims to analyze existing preclinical and clinical data on CAR T‐cell therapy in MG, with particular attention to early safety findings. By summarizing current evidence and identifying areas that require further investigation, this review seeks to provide a comprehensive understanding of this innovative therapeutic approach and its potential role in addressing unmet needs in MG management.

## 2 Search Strategy and Selection Criteria

We conducted a comprehensive literature search across PubMed, medRxiv, bioRxiv, and Google Scholar for articles published between Jan 1, 2010, and July 1, 2025. Search terms included “CAR T‐cell,” “chimeric antigen receptor,” “myasthenia gravis,” and “treatment AND myasthenia gravis.” The search was restricted to English‐language publications and encompassed a range of study types, including clinical trials, mechanistic studies, case reports, case series, and review articles.

## 3 CAR T‐Cells in B‐Cell Malignancies‐Fundamentals and Experience

**FIGURE 1:**
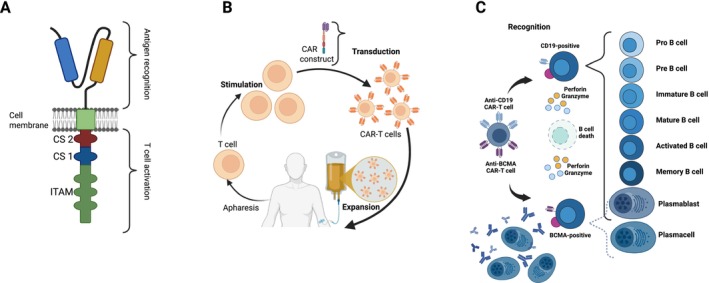
Design, production, and mode of action of CAR T‐cells. (A) A second‐generation chimeric antigen receptor (CAR) includes an antibody‐derived antigen recognition domain, a transmembrane domain anchoring it in the T cell membrane, and an intracellular signaling domain. The latter contains a costimulatory module (e.g., CS1 or CS2, representing CD28 or 4‐1BB) and the CD3ζ chain with immunoreceptor tyrosine‐based activation motifs (ITAM) essential for T cell activation. (B) To produce CAR T‐cells, patients first undergo lymphapharesis to collect peripheral blood mononuclear cells. Autologous T cells are then isolated and stimulated in vitro, typically through engagement of the T cell receptor or co‐stimulatory molecules such as CD28. These cells are genetically modified‐most often via viral vector transduction‐to express the CAR construct. The engineered T cells are subsequently expanded and reinfused into the patient following lymphodepleting chemotherapy, usually with fludarabine and cyclophosphamide. The entire manufacturing process typically takes up to 4 weeks. (C) Following infusion, CAR T‐cells recognize tumor‐associated antigens such as CD19 expressed throughout various stages of B‐cell development or BCMA (B‐cell maturation antigen), which is prevalent in malignant plasma cells. Upon antigen engagement, CAR T‐cells become activated and destroy the target cells by releasing cytotoxic molecules such as perforin and granzyme.

Autologous CAR T‐cells are a novel therapeutic approach in which patient‐derived T cells are genetically engineered to express synthetic antigen receptors combining antibody‐derived antigen‐binding domains with intracellular activation and costimulatory domains such as CD3ζ, CD28, or 4‐1BB [15, 17] (Figure 1A). The most established CAR T‐cell targets in B cell malignancies are CD19 and B‐cell maturation antigen (BCMA): CD19 is broadly expressed across B cell malignancies and restricted to B lineage cells in normal tissues, whereas BCMA is selectively expressed on mature plasma cells, making it an ideal target in multiple myeloma (Figure 1C) [20, 28]. Anti‐CD19 CAR T‐cells have demonstrated substantial efficacy in aggressive B cell lymphomas, with complete remission rates of approximately 53% in relapsed or refractory large B cell lymphoma [14, 29, 30]. BCMA‐directed CAR T‐cells achieve response rates of 73%–100% in multiple myeloma, including an overall response rate of 92% with bispecific BCMA/CD19 CAR T‐cells [28, 31].

Manufacturing of autologous CAR T‐cells involves lymphocyte apheresis followed by genetic modification, ex vivo expansion, and quality control, typically requiring around 2 weeks [32] (Figure 1B). Recent advances have reduced production times to 10 days or even 24 h without T‐cell activation or expansion, potentially lowering costs and improving accessibility [33]. Despite these advances, median time from enrollment to infusion in clinical trials remains 17–45 days, as reported in the ZUMA‐1 and ELIANA trials [29, 30].

The major toxicities of CAR T‐cell therapy are CRS, caused by systemic cytokine release, and ICANS, characterized by neurological symptoms including confusion, aphasia, seizures, and cerebral edema [34, 35, 36]. CRS occurs in up to 42% of patients, with severe cases being uncommon, while bispecific BCMA/CD19 CAR T‐cells show high CRS incidence (92%) but limited grade ≥ 3 events (8%) and low neurotoxicity rates (4%) [31, 37]. CRS is primarily managed with tocilizumab, with or without anakinra [38] whereas ICANS, occurring in approximately 30% of patients, is mainly treated with corticosteroids [23].

A frequent long‐term consequence of anti‐CD19 CAR T‐cell therapy is prolonged B cell aplasia, persisting for years in up to 38% of patients and often resulting in hypogammaglobulinemia and increased infection risk requiring immunoglobulin replacement [24, 27]. This reflects target specificity, as CD19‐directed CAR T‐cells deplete all B cell populations, whereas BCMA‐directed CAR T‐cells primarily eliminate plasma cells and less severely impair B cell immunity [28] (Figure 1C).

CAR T‐cells can persist long term and induce durable remissions, with detectable cells and sustained responses reported for over a decade in some patients, including long‐term remission in chronic lymphocytic leukemia [17, 24, 39].

CAR T‐cell exhaustion remains a major limitation, particularly in solid tumors, driven by persistent antigen stimulation and immunosuppressive microenvironments. It is characterized by reduced effector function and upregulation of inhibitory receptors such as PD‐1, TIM‐3, LAG‐3, TIGIT, and CTLA‐4 [40]. Strategies to counteract exhaustion include CAR design optimization and targeting pathways such as PD‐1, TOX/NR4A, TGF‐β, and CBL‐B [41].

Infectious complications are common after CAR T‐cell therapy, with early bacterial infections occurring in approximately 50% of patients and higher infection rates in multiple myeloma than diffuse large B cell lymphoma [26, 27]. Over time, infection frequency declines and shifts toward viral etiologies, with severe CRS increasing late infection risk [26].

Allogeneic “off‐the‐shelf” CAR T‐cells are being developed to overcome manufacturing delays and costs, offering immediate availability and standardized products. Gene‐editing approaches such as TALENs or CRISPR‐Cas9 disrupt the TRAC gene to prevent graft‐versus‐host disease while preserving functionality [42, 43]. Early clinical results show promising efficacy, although persistence may be reduced, potentially necessitating repeated dosing [43].

## 4 Role of B‐Cells and CAR T‐Cells in Autoimmunity and Neuroimmunological Diseases

CAR T‐cells represent a new therapeutic approach in autoimmune diseases, offering unprecedented advantages over conventional antibody‐based B cell depletion strategies through their autonomous action and superior tissue penetration capabilities [44, 45, 46]. Unlike B cell depleting antibodies such as rituximab, which often fail to achieve complete B cell depletion in secondary lymphoid organs and inflamed tissues, CAR T‐cells demonstrate remarkable efficacy in reaching and eliminating tissue‐resident B cell populations that typically escape conventional therapies [47]. Initial clinical applications in severe autoimmune diseases, including SLE, myositis, and systemic sclerosis, have demonstrated remarkable success, with patients achieving sustained, drug‐free remissions without severe adverse effects [48, 49]. These breakthrough results highlight the transformative potential of CD19 CAR T‐cell therapy with complete clinical remission, accompanied by seroconversion of disease‐associated antibodies while maintaining stable vaccination responses [19, 21].

The pathogenic role of B cells in autoimmune and neuroimmunological diseases extends far beyond simple autoantibody production, encompassing critical functions in antigen presentation and pro‐inflammatory cytokine secretion [49, 50]. In multiple sclerosis and other neuroinflammatory conditions, B cells serve as potent sources of pathogenic cytokines, particularly IL‐6, interferon‐γ, and GM‐CSF, which drive inflammatory cascades and sustain disease activity [51, 52]. The critical role of IL‐6 in B‐cell‐mediated pathogenesis has been demonstrated by studies showing that B cell depletion therapy ameliorates autoimmune disease specifically through ablation of IL‐6‐producing B cells, with therapeutic efficacy directly correlating with the elimination of these cytokine‐producing populations [52]. GM‐CSF‐producing B cells in particular have been identified as a distinct proinflammatory subset that efficiently activates myeloid cells and promotes pathogenic T‐helper 17 responses [52]. T‐bet‐high memory B cells that persist in inflammatory microenvironments and contribute to chronic disease progression [53, 54].

CAR T‐cells overcome these limitations through their unique ability to migrate to and eliminate hard‐to‐reach B cell populations in tissues, which may explain their rapid and profound therapeutic effects observed so far [55]. Sequential lymph node biopsies have demonstrated that CD19‐CAR T‐cells achieve complete depletion of both CD19+ and CD20+ B cells in secondary lymphoid organs, an effect not observed with rituximab treatment despite complete peripheral B cell depletion [55, 56]. Moreover, the therapeutic efficacy of CD19‐CAR T‐cells appears to preserve essential immune functions, as long‐lived plasma cells that lack CD19 expression continue to produce protective antibodies, maintaining humoral immunity against previously encountered pathogens [19, 21]. In contrast, BCMA‐targeted CAR T‐cells eliminate the plasma cell compartment [28]. The differential targeting of CD19 versus BCMA represents a crucial consideration in autoimmune disease treatment, where maintaining protective immunity while eliminating pathogenic B cell clones is paramount [45, 53].

The clinical application of CAR T‐cell therapy has expanded beyond SLE to include rare inflammatory muscle diseases and stiff person syndrome [48, 57]. Successful treatment of refractory antisynthetase syndrome demonstrated resolution of muscle inflammation and autoantibody disappearance [48]. This case illustrated the broad applicability of CD19‐CAR T‐cell therapy across different autoimmune diseases characterized by B‐cell‐mediated pathology. Sustained remission after B cell reconstitution supports immune system “reset” [19].

While the current data on CAR T‐cell therapy in autoimmune and neuroimmunological diseases remains limited, early results are quite promising, with expanding clinical investigations providing increasing evidence for the potential of this therapeutic approach to transform treatment paradigms from chronic immunosuppression to sustained clinical remission by single interventions [3, 48]. At the same time, these methodological constraints substantially limit interpretability with regard to efficacy, safety, and durability. Mechanistic and clinical data from a subsequent randomized phase 2b program further support selective targeting of BCMA‐expressing plasma cells while preserving broader immune competence [58]. Collectively, the *n* = 14 MG‐001 study therefore represents the current benchmark for clinical evaluation of CAR‐T therapy in MG, while remaining limited by its open‐label design and sample size [16]. However, as the field advances rapidly, questions remain about long‐term safety, optimal patient selection, and the mechanisms underlying the durable remissions observed [24]. The remarkable clinical responses achieved with relatively few reported serious adverse events suggest that CAR T‐cell therapy may indeed represent a potential paradigm shift in autoimmune disease management, though careful monitoring for CRS and other immune effector cell‐associated toxicities remains essential [23].

## 5 CAR T‐Cell: A Novel Immunotherapeutic Approach for Treatment‐Resistant MG

The therapeutic armamentarium for MG has expanded rapidly over the past decade, moving from broad immunosuppression toward increasingly precise, mechanism‐based interventions [4, 59]. Early progress was grounded in detailed pathophysiologic insights that linked specific antibody subclasses to neuromuscular‐junction injury and thereby rationalized new targets [1, 4]. Initial B cell depletion with rituximab illustrated how removal of CD20+ lymphocytes could recalibrate T cell to B cell co‐operation and dampen cytokine networks, yet also underscored the persistence of long‐lived plasma cells that escape CD20 blockade [2, 11, 56, 60]. These observations anticipated the seminal REGAIN trial, in which the terminal‐complement inhibitor eculizumab achieved rapid, durable clinical benefit by preventing membrane‐attack–complex formation at the postsynaptic membrane [10]. Its successor, ravulizumab, reproduced and prolonged these benefits while reducing infusion burden, confirming the complement cascade as a tractable axis even in earlier, nonrefractory disease [58]. Parallel work on Immunoglobulin G (IgG) recycling exposed the neonatal Fc‐receptor (FcRn) as another pivot of autoantibody homeostasis; the phase‐3 ADAPT study showed that the FcRn antagonist efgartigimod could safely induce repeated cycles of symptom relief and autoantibody reduction, with response‐guided redosing and tailoring exposure to need [11].

Although the magnitude and rapidity of clinical responses observed in reported CAR‐T cell therapy cases are striking, it remains premature to infer superiority over established therapies such as complement inhibition or FcRn blockade, which are supported by randomized controlled trials with long‐term outcome data [10, 11, 61].

B‐cell‐directed cell therapy now promises a deeper and possibly sustained immune reset [45]. A proof‐of‐concept case demonstrated that a single infusion of a fully human anti‐CD19 CAR T product following conventional lymphodepletion drove dramatic clinical and serological remission in refractory, anti‐AChR‐positive MG [3]. Another report demonstrated that bispecific BCMA/CD19 CAR T‐cells may eliminate pathogenic AChR antibodies in a patient with MG, converting an inability to ambulate to mountain hiking capacity within weeks [18]. Complementing these clinical anecdotes, single‐cell transcriptomic profiling revealed that BCMA‐targeted CAR T therapy reshapes T‐cell clonotypes and reconstitutes naïve B cell pools while suppressing inflammatory myeloid circuits, offering mechanistic clues to such swift, durable responses [9] (Table 1).

**TABLE 1:** Summary of recent case reports and case series on the use of CAR T‐cell therapy in patients with MG.

Authors	Year	Product	Cell count	Max. T‐cell expansion (d)	Last measured T‐cell value (d)	Lymphodepletion	Cohort size	Patient characteristics, Sex, age (years)	MG‐specific antibody	Relevant comobidities
Haghikia et al. [3]	2023	Anti‐CD19	1 × 10^8^	16	62	Cyclophosphamide 300 mg/m^2^ Fludaribine 30 mg/m^2^	1	F, 34	Anti‐AChR	—
Motte et al. [22]	2024	Anti‐CD19	1 × 10^8^	(1) 9 (2) 8	(1) 154 (2) 94	Cyclophosphamide 300 mg/m^2^ Fludaribine 30 mg/m^2^	2	(1) F, 33 (2) F, 46	(1) Anti‐AChR (2) Anti‐AChR	LEMS LEMS
Haghikia et al. [62]	2024	Anti‐CD19	1 × 10^8^	22	120	Cyclophosphamide 300 mg/m^2^ Fludaribine 30 mg/m^2^	1	F, 37	Anti‐AChR	ACPA‐positive rheumatoid arthritis
Tian et al. [9]	2024	Anti‐BMCA	(1) 6.2 × 10^7^ (2) 5 × 10^7^	(1) 10 (2) 10	(1) Approx. 90 (2) Approx. 180	Cyclophosphamide 500 mg/m^2^ Fludaribine 30 mg/m^2^	2	(1) F, 33 (2) F, 60	(1) Anti‐AChR (2) Anti‐MuSK	— —
Zhang et al. [18]	2024	Anti‐CD19/BMCA	1 × 10^6^	14	28	Cyclophosphamide 300 mg/m^2^ Fludaribine 30 mg/m^2^	1	M, 64	Anti‐AChR	Thymoma

Abbreviations: BCMA, B‐cell maturation antigen; CD19, cluster of differentiation 19; d, day; F, female; M, male.

Importantly, all currently available clinical data derive from small, uncontrolled case reports and case series comprising a total of seven patients [3, 18, 22]. While these reports consistently describe marked short‐term clinical improvement, the absence of control groups precludes reliable conclusions regarding treatment efficacy beyond individual proof‐of‐concept [44, 45].

CAR T‐cell expansion and persistence demonstrate favorable kinetics in MG patients. Analysis of published cases reveals that maximal CAR T‐cell expansion occurs between Days 8–22 postinfusion, with most patients achieving peak levels around Days 10–16 (Table 1) [3, 9, 18]. Reported CAR T‐cell persistence ranged from 28 to approximately 180 days; however, these time points represent last documented measurements rather than definitive persistence endpoints. Systematic long‐term follow‐up beyond 6 months is currently lacking, limiting conclusions regarding the durability of immune modulation and long‐term remission stability [44, 45]. The safety profile appears more favorable than in oncological applications, with adverse events consisting predominantly of low‐grade CRS (Grade 1–2), rare ICANS, and mild hematological toxicities [3, 18, 22] (Table 2). Notably, no severe life‐threatening complications have been reported in case series to date, suggesting improved tolerability in the autoimmune disease setting [44, 45].

**TABLE 2:** Reported adverse events of CAR T‐cell therapies in published cases of patients with MG.

Authors	Year	ICANS	CRS	Transaminitis	HB	Prolonged leukopenia	Prolonged lymphopenia	Kidney parameters	Thrombocytopenia	Infections	Hypogamma globulinemia	Cardiotoxicity	GI adverse effects	Fatigue
Haghikia et al. [3]	2023	−	+ (G1)	+ (G1)	N/A	−	+	N/A	N/A	−	N/A	N/A	N/A	N/A
Motte et al. [22]	2024	+ (G1)—	+ (G2) + (G1)	N/A N/A	N/A N/A	— —	N/A N/A	N/A N/A	N/A N/A	— —	N/A N/A	N/A N/A	N/A N/A	N/A N/A
Haghikia et al. [62]	2024	−	+ (G1)	N/A	+^a^	−	N/A	N/A	N/A	—	N/A	N/A	N/A	N/A
Tian et al. [9]	2024	— —	+ (G1) —	N/A	N/A N/A	— —	N/A N/A	N/A N/A	N/A N/A	Asymptomatic CMV detection	N/A N/A	N/A N/A	N/A N/A	N/A N/A
Zhang et al. [18]	2024	−	−	N/A	N/A	−	N/A	N/A	N/A	+ (Conjunctivitis/upper respiratory tract)	N/A	N/A	+	N/A

Abbreviations: CMV, cytomegalovirus; CRS, cytokine release syndrome; G, grade; GI, gastrointestinal; HB, hemoglobin; ICANS, immune effector cell‐associated neurotoxicity syndrome; N/A, not applicable.

^a^ Preexisting.

Interpretation of these findings is further complicated by challenges in defining treatment refractoriness and applying this concept consistently at the case level. In MG, refractoriness is not a single uniform entity but reflects persistent clinical activity, inability to taper immunotherapy without relapse, dependence on chronic rescue therapy, and/or intolerance to otherwise effective treatments [4, 59]. In the currently published CAR T‐cell reports, prior treatment exposure is heterogeneous, and in several patients not all mechanism‐based biologics were trialed before CAR T‐cell therapy. This heterogeneity limits inference regarding optimal positioning of CAR T‐cell therapy within the treatment algorithm and underscores the need for prospective studies employing predefined refractory criteria [44].

Taken together, the growing field of cell‐based interventions opens the door to more personalized treatment approaches, requiring the balance of efficacy with preservation of humoral immunity, the refinement of biomarkers to predict durable remission, and the definition of optimal sequencing among complementary strategies to achieve lasting, drug‐free quality of life for patients [25, 44, 45].

## 6 Challenges in CAR T‐Cell Therapy for MG

At present, no evidence‐based criteria for optimal patient selection can be derived [44, 45]. The heterogeneity of antibody status, disease duration, and prior immunotherapies across reported cases underscores the need for prospective trials with predefined stratification parameters [3, 18, 22].

Side effects such as CRS, neurotoxicity and hematotoxicity pose potential risks‐even in patients with MG [23, 24]. Standard lymphoma trials showed CRS in > 80% and grade‐3 neurotoxicity in up to 28% after CD19‐CAR T infusion [29, 63]. Pre‐existing systemic inflammation and neurological impairments (e.g., respiratory muscle weakness) can influence the tolerability of the therapy [23, 32]. Trial registries for autoimmune cellular therapies therefore recommend a meticulous pre‐diagnostic evaluation that captures comorbid infection, pulmonary reserve and bulbar function before lymphodepletion is even considered [32, 44]. In central nervous system lymphoma cohorts, patients with higher baseline inflammation were over‐represented among those who developed severe ICANS, underscoring the importance of such stratification [36, 37].

Electrophysiological, imaging, and other types of monitoring beyond clinical symptoms and signs are important in MG, as classic scales may be inadequate in severely impaired patients [1, 59]. Application of the *Immune Effector Cell‐Associated Encephalopathy* (ICE) score beside continuous EEG was able to detect subclinical seizures in almost half of the patients whose formal ICANS grade had remained 0–1, suggesting that conventional grading alone is insufficient once neuromuscular weakness complicates the examination [35, 36]. The choice of target antigen is crucial: CD19‐positive B cells play a greater role in MG than CD20‐positive ones [2, 60]. Clinical responses in SLE and other antibody‐driven disorders are more pronounced after CD19 than after CD20‐directed depletion, and early MG case reports mirror this pattern, making CD19 better suited than CD20 for a cytotoxic cassette [3, 47].

Combination therapies with T‐cell‐suppressive drugs (such as mycophenolate mofetil) must be timed to avoid double immunosuppression [3, 21, 22, 47]. EULAR–EBMT guidance allows low‐dose prednisone up to 7.5 mg 1 day prior to apheresis but advises tapering cell‐cycle blockers 4 weeks before lymphodepletion so that transduced T‐cells expand without pharmacologic brakes [32, 44]. When collecting T cells for CAR T‐cell manufacturing in patients with a history of prolonged immunosuppressive therapy‐such as those with MG‐their altered immune profile should be carefully evaluated, as pre‐existing T cell exhaustion may impact CAR T‐cell efficacy [32, 41]. Memory‐rich, less‐exhausted leukapheresis products predict deeper remissions, whereas chronic exposure to calcineurin inhibitors or mTOR blockers increases TOX‐ and NR4A‐driven exhaustion signatures that can blunt CAR proliferation [40, 41, 64, 65].

The use of chimeric autoantibody receptor (CAAR) T‐cells is currently under development and offers the potential for fewer side effects due to a lack of B cell depletion. In pre‐clinical work, AChR‐CAAR T‐cells selectively eliminated anti‐AChR B cell clones without attacking naïve pools, and similar NMDA‐CAAR constructs proved safe in encephalitis mouse models [66, 67]. However, there is no clear correlation between the clinical response and antibody titer in seropositive patients with MG, reminding clinicians that clinical monitoring, not serology, must guide decisions [1, 68]. This could be due to T‐cell–B‐cell interaction or antibody‐independent effects and might potentially diminish the efficacy of CAAR T‐cells in MG [4, 68].

Close interdisciplinary collaboration between neurology and hematology is essential for safe implementation of CAR T cell therapy in MG [44, 45]. Consensus position papers now insist on shared long‐term medical follow‐up that records neuromuscular scores beside hematological safety for at least 10 years after cell infusion, reflecting the chronicity of both immune dysregulation and delayed oncogenic risk [24, 44].

## 7 Conclusion and Outlook

The emerging field of CAR T‐cell therapy in MG represents a transformative therapeutic approach that has demonstrated considerable promise in early clinical investigations. Current evidence from multiple case reports and series indicates that targeted B‐cell depletion through anti‐CD19, anti‐BCMA, and bispecific CAR T constructs can achieve profound clinical improvements in refractory MG patients who have exhausted conventional therapeutic options. The published case series encompass seven patients across five independent reports and demonstrate consistent patterns of clinical benefit following CAR T‐cell therapy, with remarkable clinical responses that go well beyond what is attainable with conventional B‐cell‐depleting antibodies.

The differential targeting strategies each offer distinct advantages and limitations, with CD19‐directed CAR T‐cells providing comprehensive B‐cell depletion while preserving protective immunity, whereas BCMA‐targeted approaches specifically eliminate pathogenic plasma cells but may compromise broader humoral immunity. An important mechanistic limitation of CD19‐directed CAR T‐cell therapy in MG is its inability to directly target long‐lived plasma cells, which typically lack CD19 expression. As a consequence, autoreactive antibody production persists or re‐emerges despite profound B‐cell depletion, potentially limiting therapeutic durability and contributing to relapse risk. In contrast, BCMA‐directed CAR T‐cell approaches directly eliminate plasma cells and may therefore offer more sustained suppression of pathogenic autoantibodies, albeit at the cost of broader humoral immune impairment, with emerging concerns regarding rare but serious neurotoxicities, including Parkinsonism associated with anti–B‐cell maturation antigen CAR T‐cell therapy [69].

Additionally, emerging CAAR T technology offers antigen‐specific B‐cell elimination without pan‐B‐cell depletion, though it does not address antibody‐independent pathomechanisms that may contribute to disease pathogenesis in some MG patients [66, 67]. The safety profile observed in MG patients appears favorable compared to oncological applications, with predominantly grade 1–2 CRS, though specialized monitoring protocols remain essential due to the unique vulnerability of MG patients to neurological complications [23, 36]. While early clinical experiences suggest that CAR T‐cell therapy can induce profound short‐term disease control in refractory MG, it remains uncertain whether these responses reflect durable remission or transient immunological suppression. Definitive assessment of long‐term benefit awaits data from ongoing prospective trials [25].

The robust pipeline of seven registered clinical trials investigating CAR T‐cell therapy in MG, encompassing phase I through phase IIb investigations with a combined enrollment target exceeding 260 patients, reflects growing confidence in this therapeutic modality. Completion of these trials, expected between 2026 and 2029, will establish definitive evidence regarding efficacy, safety, and patient selection criteria (Table S1) [44]. Critical research priorities include establishing long‐term durability of remission, determining optimal timing within the treatment algorithm, and advancing manufacturing optimization including allogeneic “off‐the‐shelf” products [42, 43].

The seven ongoing clinical trials with results expected between 2026 and 2029 will be critical to determine durability of response, comparative efficacy, and long‐term safety. Until these data are available, CAR T‐cell therapy in MG should be regarded as an experimental, hypothesis‐generating approach rather than a standard of care. Successful integration of CAR T‐cell therapy into MG management requires addressing practical challenges including comprehensive pre‐treatment assessment protocols, careful management of concomitant immunosuppressive medications, and establishment of long‐term monitoring protocols for delayed toxicities and immune reconstitution [24, 32, 44]. If current promising signals are confirmed in larger controlled studies, CAR T‐cell therapy may fundamentally transform the treatment paradigm for refractory MG, offering the prospect of achieving durable, drug‐free remission through a single intervention [19, 45].

CAR T‐cell therapy has emerged as a highly promising therapeutic modality for refractory MG, with early clinical evidence supporting both efficacy and acceptable safety profiles [3, 16]. As the field advances through expanding clinical trials, CAR T‐cell therapy may represent the long‐awaited breakthrough toward curative treatment for patients with severe, treatment‐refractory MG, requiring continued interdisciplinary collaboration and refined patient selection strategies for successful implementation [44, 45].

## Author Contributions

**Tobias Hegelmaier:** conceptualization, writing – original draft, methodology, validation, visualization, writing – review and editing, software. **Alexander Duscha:** methodology, validation. **Christiane Desel:** review and editing. **Sarah Stassen:** methodology. **Tim Krzemien:** methodology. **Ann‐Katrin Hennemann:** methodology. **Vaia Pappa:** wiritng and edting. **Aiden Haghikia:** conceptualization, methodology, validation, visualization, investigation, writing – original draft.

## Conflicts of Interest

The authors declare no conflicts of interest.

## Supporting information


**Table S1:** Ongoing or registered clinical trials investigating CAR T‐cell therapy in MG.

## Data Availability

Data sharing not applicable to this article as no datasets were generated or analysed during the current study.
