# Supplementary material for: Chimeric Antigen Receptor T‐Cells in Myasthenia Gravis: Advances, Safety Challenges, and Future Directions
Source: Muscle Nerve. 2026 Apr 10;73(6):942–51. doi: 10.1002/mus.70222 (PMC13138368; doi:10.1002/mus.70222)
Supplement: Supplementary file 1 — Table S1: Ongoing or registered clinical trials investigating CAR T‐cell therapy in MG. [file MUS-73-942-s001.docx]

**Supplementary Table: Ongoing or registered clinical trials investigating CAR T-cell therapy in MG.**

|  | CAR T-cell product | Disease | Study design | Number of patients | Main inclusion criteria | Main results | Status |
| --- | --- | --- | --- | --- | --- | --- | --- |
| NCT04146051 | BCMA rCAR-T | Myasthenia gravis | Phase 1b/ 2a, open-label, Multicenter study | 14 | MG-ADL >6  Age > 18 years | No dose-limiting toxicity, cytokine release syndrome (no increase of IL6, IL3, TNF), or neurotoxicity was observed, adverse events: headache (6/14), nausea (5/14, vomiting (3/14), fever <24h post-infusion (4/14); improvement from baseline to week 12 in MG-ADL (-11 to -3), QMG (-19 to -9) | Published 2023 |
| NCT06220201 | CD19-CAR-T | Refractory myasthenia gravis, multiple sclerosis (RMS, PMS) | Phase 1, open-label, Multicenter Study | 120 | Age 18 to 60 years, MGFA Class of II to IV, positive antibodies against AChR or MuSK, treatment refractory (disease activity on at least 2 immunosuppressants) | …  *Primary outcome:* Number of participants with adverse events, laboratory test result abnormalities, dose-limiting toxicities up to week 104 | Announced March, 2024; estimated completion July, 2027 |
| NCT06193889 | CD19-CAR-T | Refractory myasthenia gravis | Phase 2, open-label, Multicenter Study | 20 | Age 18 to 75 years, positive antibodies against AChR and MuSK, MGFA Class of IIB to IV, MG-ADL ≥ 6, Failed treatment ≥ 1 year with ≥ 2 immunosuppressants or failure of at least 1 immunosuppressive therapy and required chronic plasmapheresis or IVIG | …  *Primary outcome:* Incidence of adverse events and laboratory abnormalities within 2 years; improvement in MG-ADL within 24 weeks | Announced August, 2024; estimated completion May, 2027 |
| NCT06626919 | BCMA-CAR-T | Myasthenia gravis | Phase 1, open-label, Multicenter Study | 30 | Age >18 years, MGFA Class of II to IVa, clinically active disease requiring ongoing therapy, MG-ADL 6, QMG >10, positive specific antibodies | …  *Primary outcome:* Type, incidence, and severity of treatment-emergent adverse events including dose-limiting toxicity and laboratory abnormalities up to 24 months, Evaluation of the maximum tolerated dose to establish the recommended phase 2 dose | Announced January, 2025; estimated completion June, 2028 |
| NCT06359041 | BCMA-CAR-T | Myasthenia gravis | Phase 1-2, open label, Multicenter Study | 12 | Age 18 to 70 years, MGFA class II, III, IVa, and IVb; seropositive (antibodies AChR, MuSK and/or LRP-4) or seronegative MG | …  Primary outcome: To evaluate incidence and severity of adverse events up to 28 days after infusion | Announced December, 2024; estimated completion September, 2029 |
| NCT04146051 | BCMA-CAR-T | Myasthenia gravis | Phase 1b-2b, randomized, quadruple-blinded, Multicenter study | 30 | Age ≥ 18, seropositive and seronegative patients included | …  Primary outcome: Proportion of patients with MGC-score improvement of ≥5 points within 85 days, part 3: comparison of the effect between Descartes-08 and placebo by change in MGC-score from baseline to week 12 | Announced December, 2019; estimated completion March, 2026 |
| NCT06485232 | CD19-BCMA CAR-T | Refractory autoimmune diseases of the nervous system | Early Phase 1, open label, Single-center study | 25 | Age 18-75 years (for MS patients, 18-55 years), refractory neurological autoimmune diseases who have failed standard treatment | …  *Primary outcome:* Incidence of dose-limiting toxicities first 28 days after infusion, incidence of adverse events up to 12 months after infusion | Announced February, 2025; estimated completion December, 2027 |
| NCT06371040 | CD19-BCMA CAR-T | Refractory myasthenia gravis | Phase 1, open-label, Single-center study | 12 | Age 18 to 80 years, positive antibodies against AChR, MuSK or LRP-4, evaluated as refractory MG | …  *Primary outcome:* Frequency, type, and severity of adverse events occurring within 4 weeks | Announced July, 2024; estimated completion December, 2026 |

This table lists currently ongoing, completed, or registered clinical trials evaluating the use of CAR T-cell therapy in patients with MG. The table includes information on study identifiers, number of patients, recruiting status, target antigen (e.g., CD19 or BCMA), study phase, and primary endpoints. Trial data were obtained from public registries such as ClinicalTrials.gov and the WHO International Clinical Trials Registry Platform.

Abbreviations: CD19 = cluster of differentiation 19; BCMA = B-cell maturation antigen; CAR = chimeric antigen receptor; MS = multiple sclerosis.
